# miR-10a-5p Inhibits the Differentiation of Goat Intramuscular Preadipocytes by Targeting KLF8 in Goats

**DOI:** 10.3389/fmolb.2021.700078

**Published:** 2021-08-13

**Authors:** Qing Xu, Yong Wang, Xin Li, Yu Du, Yanyan Li, Jiangjiang Zhu, Yaqiu Lin

**Affiliations:** ^1^ Key Laboratory of Qinghai-Tibetan Plateau Animal Genetic Resource Reservation and Utilization of Education Ministry, Southwest Minzu University, Chengdu, China; ^2^ Key Laboratory of Qinghai-Tibetan Plateau Animal Genetic Resource Reservation and Exploitation of Sichuan Province, Southwest Minzu University, Chengdu, China; ^3^ College of Animal Science and Veterinary, Southwest Minzu University, Chengdu, China

**Keywords:** MiR-10a-5p, KLF8, goat, intramuscular preadipocytes, differentiation

## Abstract

Intramuscular fat contributes to the improvement of meat quality of goats. MicroRNAs (miRNAs) have been reported to regulate adipocyte differentiation and maturation. The aim of our study was to clarify whether miR-10a-5p regulates goat intramuscular preadipocyte (GIPC) differentiation and its direct downstream signaling pathway. GIPCs were isolated from longissimus dorsi, whose miR-10a-5p level was measured at different time point of differentiation induction. Adipogenic differentiation of the GIPCs was evaluated by Oil Red O and BODIPY staining, and the expression changes of adipogenic genes like ACC, ATGL, CEBPβ, PPARγ, etc. Related mechanisms were verified by qPCR, a bioinformatic analysis, a dual-luciferase reporter assay, overexpression, and siRNA transfection. Oil Red O and BODIPY staining both with adipogenic gene detection showed that miR-10a-5p suppressed the accumulation of lipid droplets in GIPCs and inhibited its differentiation. The dual-luciferase reporter assay experiment revealed that miR-10a-5p regulates GIPC differentiation by directly binding to KLF8 3’UTR to regulate its expression. Thus, the results indicated that miR-10a-5p inhibits GIPC differentiation by targeting KLF8 and supply a new target for fat deposition and meat quality improvement.

## Introduction

Intramuscular fat (IMF) is deposited in skeletal muscle fibers (Hocquette et al., 2010). IMF can be used to store energy and functions during exercise, but an excessive accumulation of IMF in the muscle is related to many diseases in people, such as diabetes, insulin resistance, lipodystrophy, etc. (Goodpaster et al., 2000; Shoelson et al., 2007; Tada et al., 2020; Yamada et al., 2020). The IMF content in animals is not only an important index of high quality of meat but also an important character of good germplasm. IMF is an important factor for meat quality which is connected with meat tenderness, meat flavor, and color (Wood et al., 2004; Chen and Sui, 2018; Liu et al., 2019). The number and size of intramuscular adipocytes mainly determine the IMF contents, and intramuscular adipocytes are very important because they provide sites for later marbling fat deposition. However, the mechanism of preadipocyte differentiation still needs to be further investigated.

MicroRNAs (miRNAs or miRs) are small noncoding RNAs; several studies suggested that they are the negative regulator over the process of target gene expression through degrading mRNAs or inhibiting the translation of mRNAs (Mourelatos, 2008; Li et al., 2014; Schreck et al., 2017; Lin et al., 2019; van der Kwast et al., 2020). Recently, more and more studies have proved the significance of miRNAs in regulating adipogenic differentiation. However, roles of miRNAs in GIPC differentiation shift fate are still unclear.

In the current study, we explored the action of miR-10a-5p in GIPC differentiation as well as mechanisms during the process. In general, we demonstrated that miR-10a-5p expression was significantly changed during preadipocyte differentiation in goats. MiR-10a-5p inhibits GIPC differentiation by targeting KLF8. Therefore, our conclusion supplied a new idea and theoretical basis for the basic research of improving the quality of meat.

## Materials and Methods

### Isolation and Cell Culture of Goat Intramuscular Preadipocytes

The 7-day-old Jianzhou Daer male goats (*n* = 3) were used as an experimental model. The GIPCs were isolated and cultured, as we previously described (Xu et al., 2018; Ma et al., 2021). Briefly, the isolated longissimus dorsi muscles of goats were washed with PBS for three times and minced and then were digested with an equal volume of collagenase type II at 37°C for 2 h in a shaking water bath every 5 min. Steel mesh filters of 200 and 400 µm were utilized to isolate digested cells. The rinsed filtrated cells by DMEM/F12 medium were centrifuged twice at 2000 r/min for 5 min to collect sediment clumps and then the supernatant was discarded. The viable cells were resuspended in DMEM/F12, including 10% fetal bovine serum and plated in 25-cm^2^ flask in a 5% CO_2_ atmosphere at 37°C for subsequent culture. Experimentation on goats performed in the present study had been given prior approval by the Ethics Committee of Southwest Minzu University under permit no. SMU20160108, and all of the methods were performed according to the guidelines and regulations.

### Preadipocyte Differentiation Induction

We induced GIPCs adipogenic differentiation *in vitro* as before (Xu et al., 2018). Briefly, adipogenesis induction medium [MEM/F12 containing 10% FBS and 50 μmol•L^-1^ oleic acid (Sigma)] was used to culture GIPCs in 12-well plates with a density of 1 × 10^6^ cells per well for the required time point. We changed the culture medium every other day. After induction, Oil Red O staining and BODIPY staining were used to distinguish mature adipocytes from preadipocytes during the process of culture.

### Oil Red O and BODIPY Staining

Adipogenic differentiation of the GIPCs was assessed by Oil Red O or BODIPY staining as previously described (Xu et al., 2018). The GIPCs were washed with PBS and fixed in 10% formaldehyde for 10 min, then washed with PBS, and stained using the Oil Red O or BODIPY working solutions for 20 min. The cells were then observed and photographed after washing. After photographing, the cells were destained in 1 ml 100% isopropanol for 15 min and the Oil Red signal was quantified by measuring the absorbance at 490 nm (OD 490) as a semi-quantitative assessment method to determine the extent of differentiation. The stained area of Oil Red O or BODIPY staining was measured using ImageJ (NIH, Bethesda, MD, United States).

### qRT-PCR

Total RNA from cells was extracted using the TRIzol reagent (TaKaRa) according to the manufacturer’s protocol. The mRNAs were reverse transcribed using the RevertAid First Strand cDNA Synthesis Kit (Thermo) according to the protocol. Then, amplification reactions were performed using amplification primers with the SYBR Green PCR Master Mix (TaKaRa); the reaction volumes were 20 μl. Then 1 μl of cDNA was applied in every set of experiment. The mRNA expression levels were standardized to UXT or U6. Information on primers for qPCR is listed in Table 1.

**TABLE 1:** The sequences information of specificity primers.

Gene/miRNA (Accession number in GenBank)	Sequence
*ACC* (XM_018064169.1)	GGA​GAC​AAA​CAG​GGA​CCA​TT
	ATCAGGGACTGCCGAAAC
*ATGL* (NM_001285739.1)	GGT​GCC​AAT​ATC​ATC​GAG​GT
	CACACCCGTGGCAGTCAG
*AP*2 (NM_001285623.1)	TGA​AGT​CAC​TCC​AGA​TGA​CAG​G
	TGA​CAC​ATT​CCA​GCA​CCA​GC
*CEBP*α (XM_018062278)	CCG​TGG​ACA​AGA​ACA​GCA​AC
	AGG​CGG​TCA​TTG​TCA​CTG​GT
*CEBP*β (XM_018058020.1)	CAA​GAA​GAC​GGT​GGA​CAA​GC
	AACAAGTTCCGCAGGGTG
*DGAT*2 (NM_001314305.1)	CAA​TAG​GTC​CAA​GGT​AGA​GAA​GC
	ACC​AGC​CAG​GTG​AAG​TAG​AGC
*GLUT*4 (NM_001314227.1)	TGC​TCA​TTC​TTG​GAC​GGT​TCT
	CAT​GGA​TTC​CAA​GCC​TAG​CAC
*FASN* (NM_001285629.1)	TGTGCAACTGTGCCCTAG
	GTCCTCTGAGCAGCGTGT
*HSL* (XM_018062484.1)	AGG​GTC​ATT​GCC​GAC​TTC​C
	GTC​TCG​TTG​CGT​TTG​TAG​TGC
*LPL* (NM_001285607.1)	TCC​TGG​AGT​GAC​GGA​ATC​TGT
	GAC​AGC​CAG​TCC​ACC​ACG​AT
*PPAR*γ (NM_001285658)	AAG​CGT​CAG​GGT​TCC​ACT​ATG
	GAA​CCT​GAT​GGC​GTT​ATG​AGA​C
*Pref*1 (KP686197.1)	CCG​GCT​TCA​TGG​ATA​AGA​CCT
	GCC​TCG​CAC​TTG​TTG​AGG​AA
*SREBP*1 (NM_001285755)	AAG​TGG​TGG​GCC​TCT​CTG​A
	GCAGGGGTTTCTCGGACT
*KLF*8 (KX247671)	GAC​TAC​AGC​AAG​AAC​CAG​CAG​C
	CTC​CTG​TAT​GGA​TTC​TGC​GGT
*UXT* (XM_005700842.2)	GCA​AGT​GGA​TTT​GGG​CTG​TAA​C
	ATG​GAG​TCC​TTG​GTG​AGG​TTG​T
*U*6 (NR_138,085.1)	TGG​AAC​GCT​TCA​CGA​ATT​TGC​G
	GGA​ACG​ATA​CAG​AGA​AGA​TTA​GC
miR-10a-5p	CAG​CTG​TAC​CCT​GTA​GAT​CCG​A
	GTGCAGGGTCCGAGGT

### Transfection

Small interfering RNA (siRNA) against KLF8, 5’-CAGACUCUUGUAGUGUCCACUUCAAdTdT-3’ was synthesized by Invitrogen. KLF8 expression plasmid was constructed by inserting expanded KLF8 cDNA (KX247671) fragments into pcDNA3.1 vector (sense primer sequence: 5’CGG​GGT​ACC​ATG​GAT​GAA​CTC​ATA​AAC​AAC​T-3’, anti-sense primer sequence: 5’-ATA​AGA​ATG​CGG​CCG​CTT​ACA​CGG​TGT​CAT​GGC​GC-3’). The KLF8 interference (designated as siKLF8) or negative control (siNC) GIPCs were constructed using siRNA. The KLF8 overexpression (designated as KLF8) GIPCs were constructed using an expression plasmid, and the control cells for the KLF8 overexpression group were designated as a vector. Cells had been pre-cultured for 2 h in a serum-free medium for transfection. Then plasmid or siRNA was introduced into the cells using a Lipofectamine 3000 transfection reagent, in accordance with the manufacturer’s instruction (Invitrogen, Carlsbad, United States).

The miR-10a-5p mimics (designated as mimics: UAC​CCU​GUA​GAU​CCG​AAU​UUG​U), an inhibitor (designated as inhibitor: ACA​AAU​UCG​GAU​CUA​CAG​GGU​A), and a respective negative control (designated as mock: UUG​UAC​UAC​ACA​AAA​GUA​CUG, NC: CAG​UAC​UUU​UGU​GUA​GUA​CAA) (Genepharma, Shanghai, China) as needed were transfected into the GIPCs by Lipofectamine 3000 (Invitrogen, Carlsbad, United States) and opti-MEM (Gibco BRL Co., LTD) culture medium according to the manufacturer’s instruction.

After 12-h transfection, the original medium was replaced by a fresh differentiation medium to induce GIPC differentiation. After 48-h induction, the cells were used for Oil red O or BODIPY staining or collected to extract RNA for qPCR detection.

### Luciferase Reporter Assay

For the luciferase reporter assay, the 3’UTR of KLF8 containing the wild or mutant miR-10a-5p target sites was cloned using primers with *Not*I and *Xho*I (Thermo, MA, United States) cleavage sites. The wild or mutant type 3’UTR fragment was inserted into the corresponding site of the psiCHECK vector and then co-transfected into 293T cells with miR-10a-5p mimics/mock. After 48 h transfection, the cells were harvested and the Dual-Luciferase Reporter Assay System Kit (Promega, Madison, WI, United States) was used for detecting dual-luciferase activity, according to the manufacturer's instructions.

### Statistical Analysis

All data were presented as “mean ± SD.” The variance of data was analyzed by SPSS 17.0, followed by Duncan’s multiple comparisons test. * indicates the *p* values were <  0.05, ≥  0.01, whereas ** indicates *p* values <  0.01. All experiments in our study were carried out for three times at least.

## Results

### miR-10a-5p Expression Changed During GIPCs Differentiation

**FIGURE 1:**
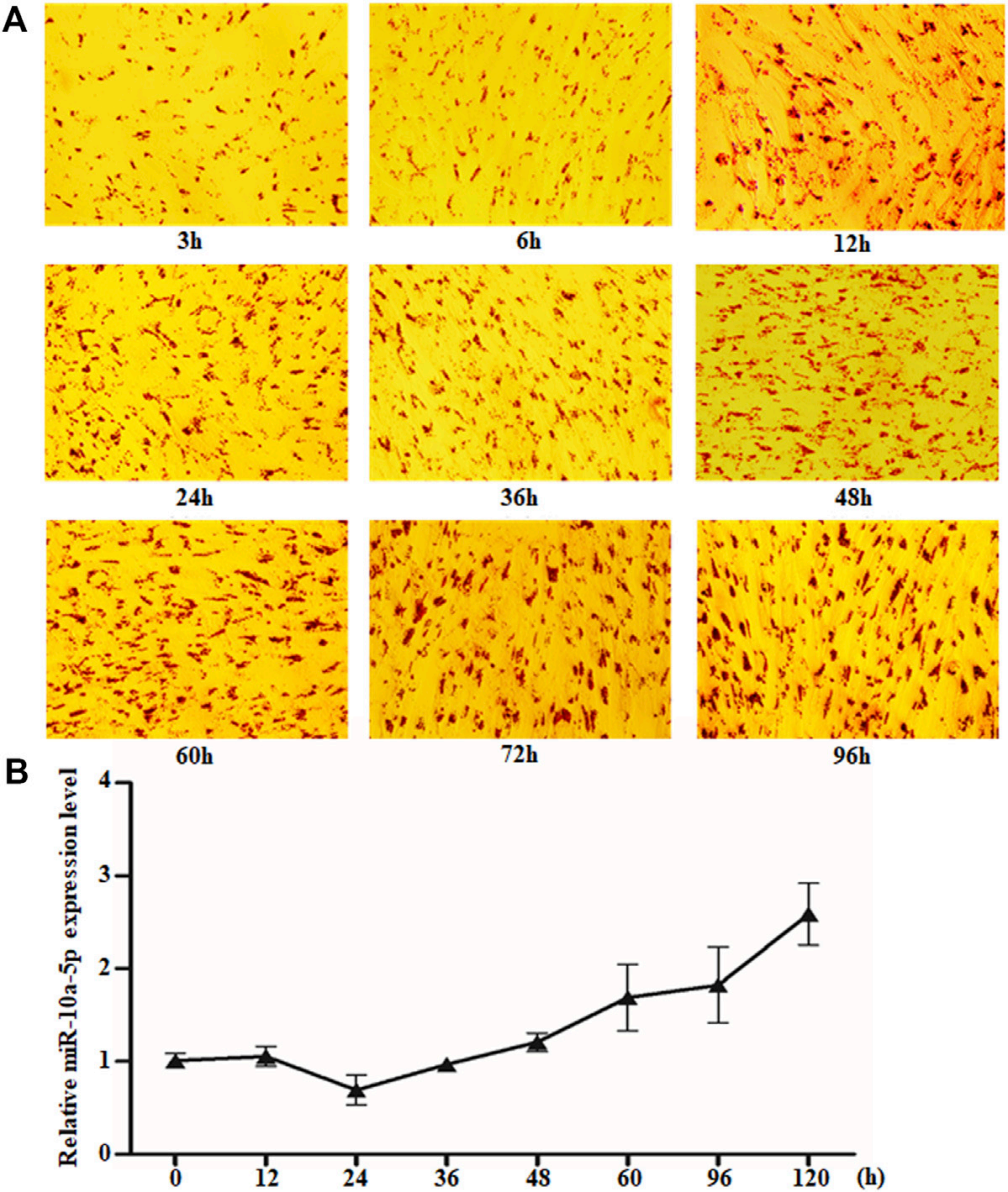
miR-10a-5p expression changed obviously during GIPC differentiation. **(A)** Representative images of Oil O staining of GIPCs cultured in oleic acid induction medium for different hours. **(B)** qRT-PCR analysis of the relative level of miR-10a-5p expression in GIPCs cultured in oleic acid induction medium for the hours as indicated. N > or = 3 for A and B.

MicroRNAs have been reported to regulate adipogenic differentiation (Hamam et al., 2015; Tang et al., 2017; Ai et al., 2019; Li et al., 2019); however, the role of miR-10a-5p in goat intramuscular adipogenesis has not been reported. The differential expression of miR-10a-5p after GIPC differentiation has been observed by miRNA sequencing technology in our previous study (data not shown). In order to clarify the role of miR-10a-5p in the differentiation of GIPCs, we first isolated intramuscular preadipocytes from goat longissimus dorsi and induced them to adipogenic differentiation. Oil red O staining was used to ascertain the extent of differentiation; our obtained results showed that the lipid droplets accumulation increased with the extension of induction time, and the GIPCs were differentiated completely after 60-h induction (Figure 1A). Then qRT-PCR was implemented to research the role of miR-10a-5p in adipogenic differentiation of GIPCs. The test result showed an obvious alteration in the expression of miR-10a-5p during differentiation when compared to 0 h (Figure 1B). All above indicate that miR-10a-5p may regulate the adipogenic differentiation of GIPCs.

### miR-10a-5p Inhibits the Adipogenic Differentiation of GIPCs

**FIGURE 2:**
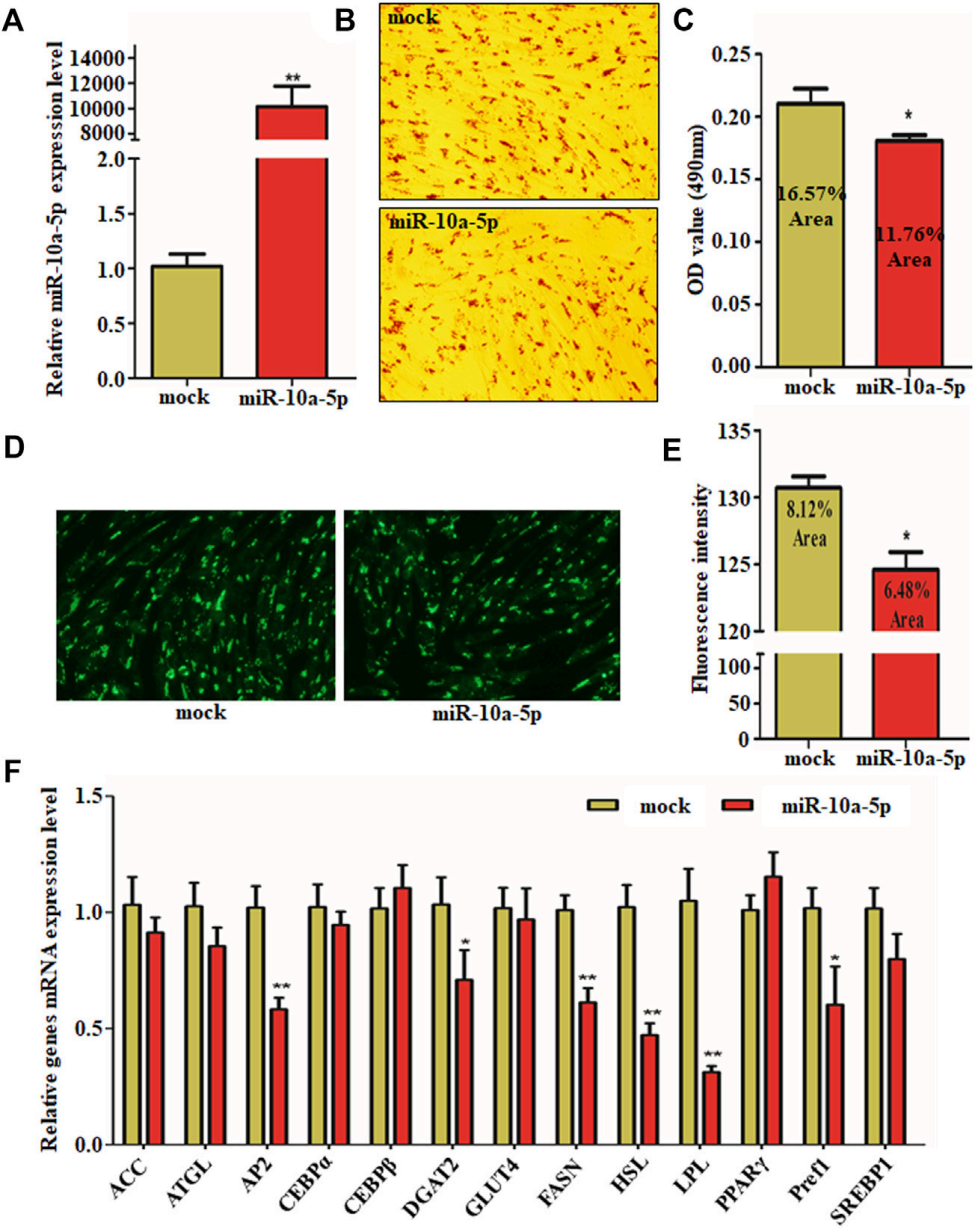
Overexpression of miR-10a-5p inhibits GIPC differentiation. **(A)** qRT-PCR analysis of levels of miR-10a-5p expression in GIPCs with mimics or control transfected for 24 h. **(B)** Representative images of Oil Red O staining of GIPCs with miR-10a-5p mimics or control and **(C)** semi-quantitative assessment of Oil Red O content absorbance detection at 490 nm. **(D)** Representative images of mature adipocytes stained with BODIPY and **(E)** stained area was measured using ImageJ. **(F)** qRT-PCR analysis of levels of genes expression in GIPCs with miR-10a-5p mimics or control. N > or = 3, * indicates *p* values  <  0.05 and ≥  0.01, ** indicates *p* values  <  0.01.

To clarify the effect of miR-10a-5p on GIPC adipogenic differentiation, miR-10a-5p mimics (named as miR-10a-5p) or its control (named as mock) was transfected to GIPCs to overexpress miR-10a-5p. The expression of miR-10a-5p in GIPCs increased ∼ 10,000 times caused by mimic transfection than the same amount of control vector transfected cells (Figure 2A). Preadipocyte differentiation is associated with lipid droplets accumulation in the cells. Oil red O and BODIPY stainings were utilized as lipid droplet detection methods. In our study, after miR-10a-5p overexpression, the lipid droplet accumulation was significantly decreased in the group of mimic-transfected cells than the control group (Figures 2B–E). Oil red O and BODIPY staining results showed about 20–30% reduction of lipid droplets with miR-10a-5p overexpression (Figures 2B–E). Additionally, there are representative of the genes upregulated during the differentiation process from preadipocytes to mature adipocytes. Indeed, the mRNA expression levels of representative genes AP2, DGAT2, FASN, HSL, LPL, and Pref1 were obviously inhibited due to the overexpression of miR-10a-5p in GIPCs (Figure 2F). All these results implied that miR-10a-5p may weaken the adipogenic differentiation of GIPCs.

**FIGURE 3:**
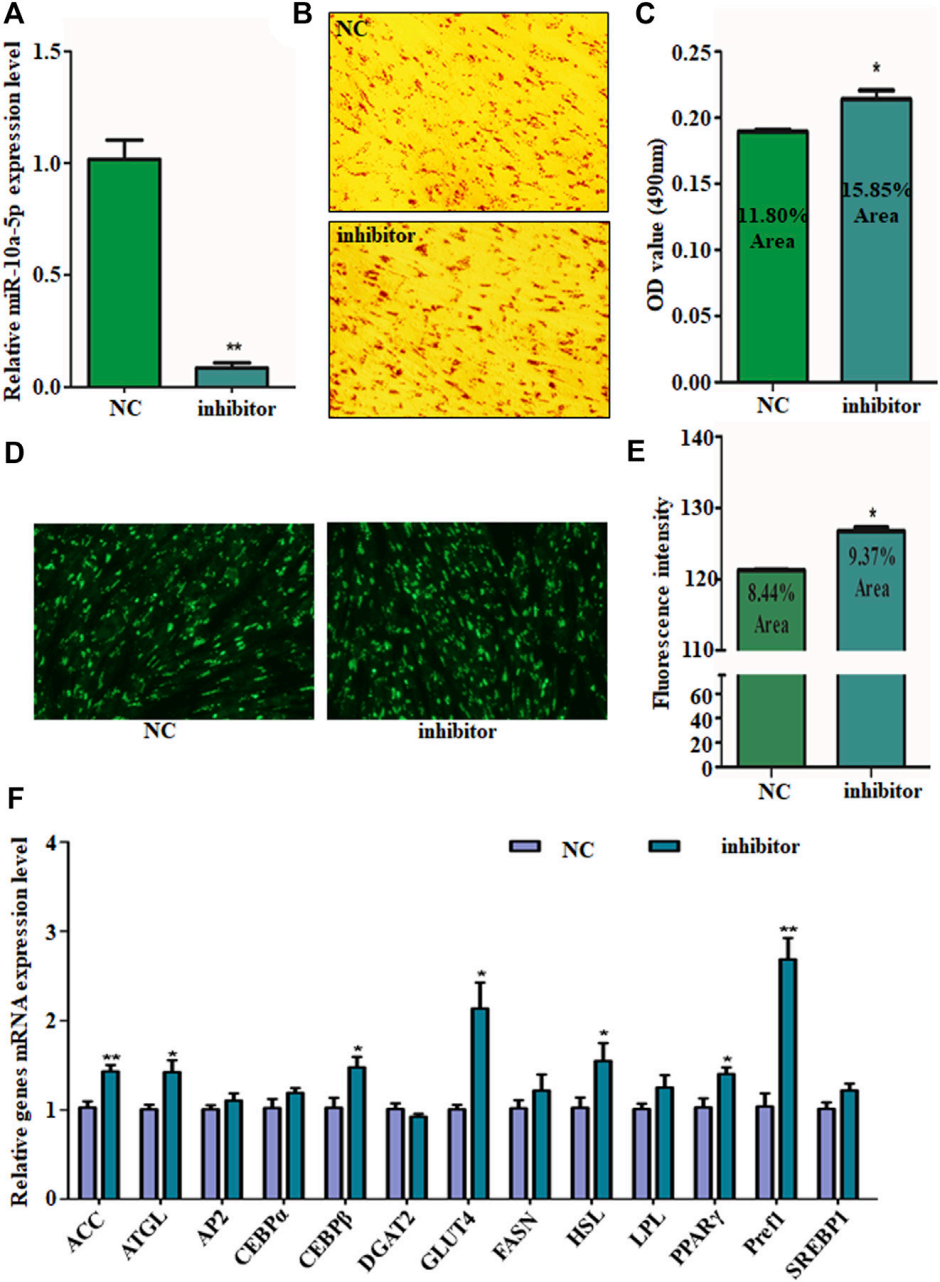
Silence of miR-10a-5p promotes GIPC differentiation. **(A)** qRT-PCR analysis of levels of miR-10a-5p expression in GIPCs with inhibitor or control transfected for 24 h. **(B)** Representative images of Oil Red O staining of GIPCs with miR-10a-5p inhibitor or control and **(C)** semi-quantitative assessment of Oil Red O content absorbance detection at 490 nm. **(D)** Representative images of mature adipocytes stained with BODIPY and **(E)** stained area was measured using ImageJ. **(F)** qRT-PCR analysis of levels of genes expression in GIPCs with miR-10a-5p inhibitor or control. N > or = 3, * indicates *p* values  <  0.05 and ≥  0.01, ** indicates *p* values <  0.01.

For further validating the suppression effect of miR-10a-5p on GIPC adipogenic differentiation, miR-10a-5p inhibitor (named as inhibitor) or its control (named as NC) was transfected to GIPCs to silence miR-10a-5p expression. About 80% of the interference efficiency was caused by inhibitor transfection compared to the NC group (Figure 3A). Oil red O and BODIPY staining results showed that compared with the control group, miR-10a-5p knockdown significantly promoted the about 10–20% of lipid droplet accumulation (Figures 3B–E). Coincidently, the mRNA levels of important markers of adipocyte differentiation like ACC, ATGL, CEBPβ, GLUT4, HSL, PPARγ, and Pref1 were upregulated due to the knockdown of miR-10a-5p in GIPCs (Figure 3F). Therefore, miR-10a-5p inhibits adipogenic differentiation of GIPCs.

### miR-10a-5p Targets the 3’UTR of KLF8 mRNA

**FIGURE 4:**
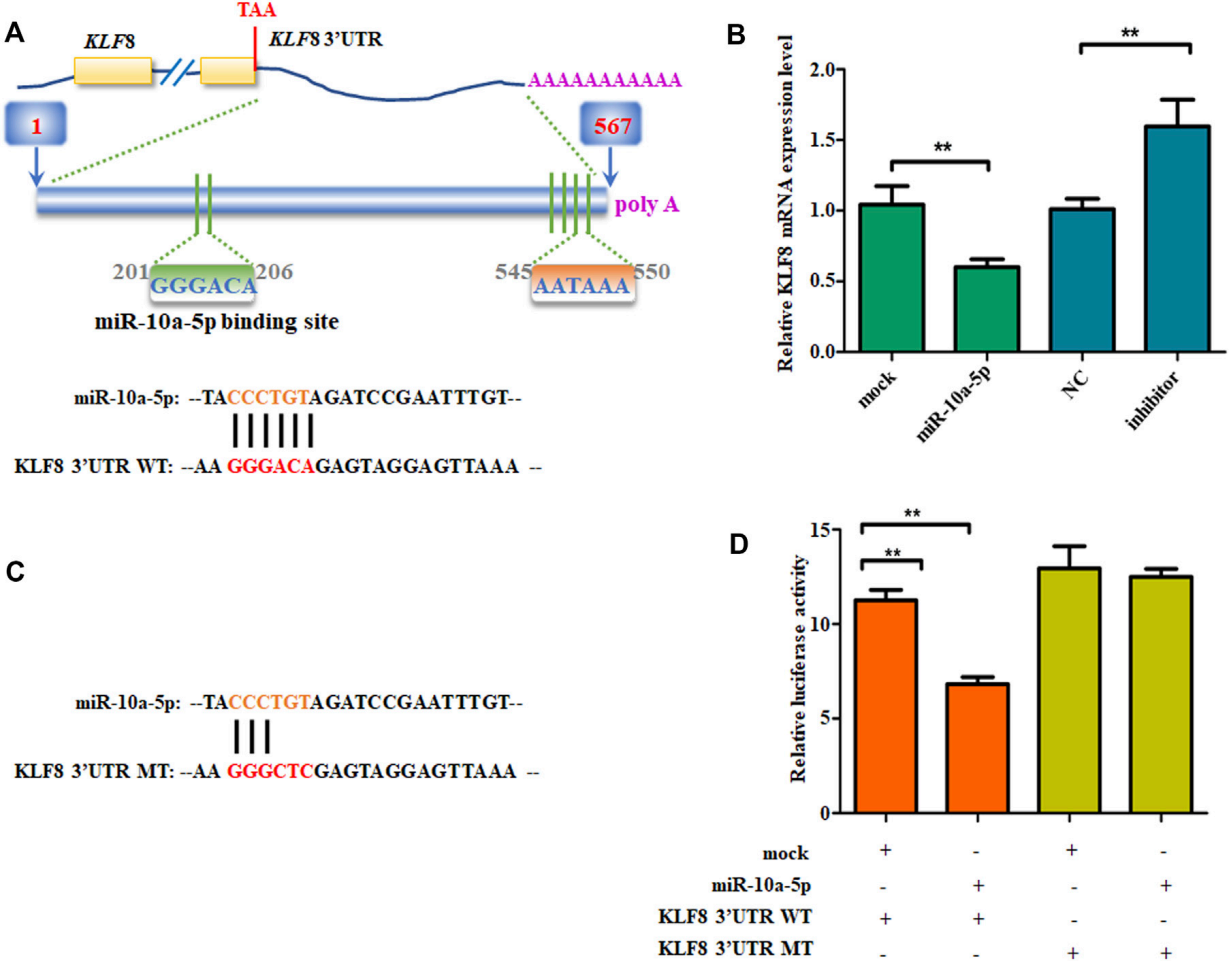
miR-10a-5p directly bind to KLF8 3’UTR. **(A)** The predicted miR-10a-5p binding site in KLF8 3’UTR. **(B)** qRT-PCR analysis of levels of genes expression in GIPCs with miR-10a-5p mimics, inhibitor, or each control. **(C)** mutant miR-10a-5p binding site in KLF8 3’UTR. **(D)** Luciferase assay of transfected with wild-type or mutant KLF8 3’UTR plasmid in 293T cells. N >or = 3 for B and D, * indicates *p* values <  0.05 and ≥ 0.01, ** indicates *p* values <  0.01.

It has been well known that miRNA binds to the 3’-UTR of target mRNA’s complementary sequences, so the target gene’s mRNA expression can be inhibited (Redis and Calin, 2017; Rouleau et al., 2017; Sun et al., 2017). TargetScan (Garcia et al., 2011), microRNAseq (Yan et al., 2018), miRDB (Wong and Wang, 2015), and DIANA-microT (Maragkakis et al., 2009) were the tools used for predicting the miR-10a-5p possible target genes. Among all the potential target genes predicted in both databases, we chose KLF8, as it is a positive regulator of 3T3-L1 differentiation and knocking down its expression can reduce RXRα overexpression-caused GIPC differentiation (Lee et al., 2012; Xu et al., 2020). Also, its family members are important regulators for adipogenic differentiation (Kinoshita et al., 2010; Pei et al., 2011; Tahmasebi et al., 2013; Shen et al., 2018; Chen J. et al., 2020). By sequence analysis, there is potential binding site of miR-10a-5p in the 3'UTR of KLF8 in goat (Figure 4A). First, we measured the mRNA level of KLF8 in miR-10a-5p mimics or inhibitor-transfected cells. As expected, the mimics of miR-10a-5p significantly downregulated the mRNA level of KLF8, while miR-10a-5p inhibitor upregulated its mRNA expression when compared with each control group, respectively (Figure 4B). In order to make sure whether miR-10a-5p can directly target KLF8 3'UTR, we mutated miR-10a-5p–binding sites in the KLF8 3’UTR area (Figure 4C). Luciferase report vectors were constructed with wild-type KLF8 3’UTR (KLF8 3’UTR WT) and mutated KLF8 3’UTR (KLF8 3’UTR MT). The KLF8 luciferase activity was measured for describing miR-10a-5p function on luciferase translation. The results showed that luciferase activity of wild-type KLF8 3’UTR was significantly inhibited by miR-10a-5p overexpression, yet mutated KLF8 3’UTR terminated this effect (Figure 4D). Taken together, we confirmed that KLF8 is the direct target of miR-10a-5p. Then, we got the conclusion that miR-10a-5p targets KLF8 and regulates KLF8 expression.

### KLF8 Promotes the Adipogenic Differentiation of GIPCs

**FIGURE 5:**
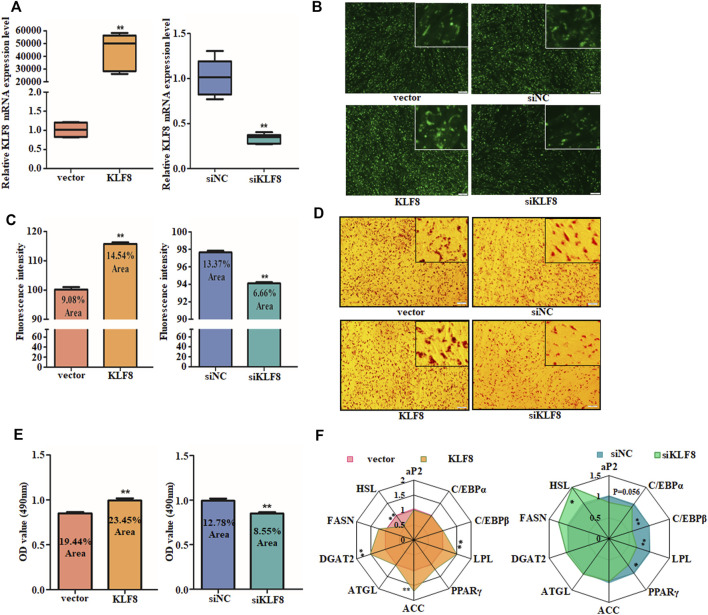
KLF8 promotes GIPC differentiation. **(A)** qRT-PCR analysis of levels of KLF8 with KLF8 siRNA, expression plasmid, or each control transfected for 24 h. **(B)** Representative images of mature adipocytes stained with BODIPY and **(C)** stained area was measured using ImageJ. **(D)** Representative images of Oil Red O staining of GIPCs with miR-10a-5p inhibitor or control and **(E)** semi-quantitative assessment of Oil Red O content absorbance detection at 490 nm. **(F)** qRT-PCR analysis of genes expression in GIPCs with KLF8 up- or downregulation, the result was showed by a network diagram. N >or = 3 for B and D, * indicates *p* values <  0.05 and ≥ 0.01, ** indicates *p* values <  0.01.

Since KLF8 is the target of miR-10a-5p and plenty of studies have shown that KLF8 and its family members are regulators of adipogenic differentiation, we further explored the relationship between KLF8 expression and GIPCs differentiation. We first synthesized KLF8 siRNA (named as siKLF8 and its control named as siNC) and constructed its expression plasmid (named as KLF8 and its control named as vector). Both the expression plasmid and siRNA were effective that cells transfected with the KLF8 expression plasmid can upregulate its expression about 5,000 times, and KLF8 siRNA can downregulate its expression about 60 times (Figure 5A). Oil red O and BODIPY staining results showed that KLF8 overexpression could contribute to the lipid droplet accumulation; nevertheless, KLF8 knockdown can inhibit the lipid droplet accumulation when compared with each control group, respectively (Figures 5B–E). qPCR was used to detect relative adipogenic gene expression, and the crucial adipocyte differentiation markers DGAT2, ACC, and LPL were markedly promoted by KLF8 overexpression (Figure 5F). Also, LPL, PPARγ, and C/EBPβ as adipocyte differentiation genes were obviously inhibited by KLF8 interference (Figure 5F). In summary, our results indicated that KLF8 confers GIPCs with more properties of adipogenic differentiation.

## Discussion

Preadipocytes differentiate into adipocytes, which improve the color and flavor of meat in animals. According to the results we obtained, we expounded that miR-10a-5p is involved in regulating GIPCs adipogenic differentiation. miR-10a-5p directly targets the 3’UTR of KLF8 to inhibit its expression which promotes adipogenic differentiation. Furthermore, miR-10a-5p inhibits the accumulation of lipid droplets and the expression of relative adipogenic genes. Our results indicate that miR-10a-5p regulates alteration and lineage fate in GIPCs at the adipogenic differentiation process.

Accumulation of lipid droplets in the cells is associated with the differentiation of preadipocytes (Lee et al., 2017; Peng et al., 2018). This was verified by Oil red O and BODIPY staining in our study. Additionally, the differentiation of adipocytes is featured with genes expression alteration such as PPARγ, LPL, HSL, and C/EBP, and so on (Rosen and MacDougald, 2006; Kim et al., 2015; Lee et al., 2017; Peng et al., 2018; Chen X. et al., 2020). Indeed, during the differentiation process from preadipocytes to mature adipocytes, ACC, ATGL, CEBPβ, GLUT4, HSL, PPARγ, and Pref1 are the representative upregulated genes. It is interesting that the adipogenic genes expression in miR-10a-5p overexpression GIPCs are not consistent with miR-10a-5p knockdown cells (Figures 2F, 3F). KLF8 overexpression and knockdown cells showed the same phenomenon (Figure 5F). Here, we confirmed that KLF8 was the target of miR-10a-5p, but the knockdown of KLF8 causing the expression tendency of differentiation-related genes was not completely consistent with miR-10a-5p overexpression (Figures 2F, 5F). Also, the expression trend of differentiation-related genes did not present the same consistency in both KLF8 overexpression cells and miR-10a-5p knockdown cells (Figures 3F, 5F). One possible explanation for this observed deviation is that the differentiation of GIPCs was regulated by more complex mechanisms, and this deviation might be referred to other regulation pathways for which further investigation would be need.

miRNAs are known as endogenous small noncoding RNAs that have been identified as gene expression post-transcriptional regulators, and miRNAs bind mainly to the target mRNA’s 3’ untranslated regions (UTRs), resulting in the blockade of mRNA translation or mRNA degradation (Kerr et al., 2011; Callegari et al., 2013). Thus, miRNAs play vital roles in the differentiation and maturation of adipocytes, as shown in the findings of the present studies (Kato et al., 2012; Kobayashi et al., 2013; Miyoshi et al., 2014; Fujita et al., 2015a; Fujita et al., 2015b; Fujihara et al., 2015; Fujimori et al., 2015; Kato et al., 2016). Several miRNAs have been reported to contribute to lipid synthesis, metabolism, transportation, and storage. miR-10a-5p was reported to be relevant to proliferation, metastasis, invasive, drug resistance, inflammation, and other behaviors in cancer cells. In a previous investigation, miR-10a-5p was shown to restrain adipogenic differentiation in primary mouse preadipocytes. In the current study, up- or downregulation of miR-10a-5p was found to be involved in the differentiation of preadipocytes in goats. We also found that miR-10a-5p binds to the 3’UTR of KLF8 to perform its functions in an KLF8-dependent pathway. Taken together, our studies show that miR-10a-5p is critical for GIPC differentiation.

## Conclusion

Our results show that miR-10a-5p acts as an inhibitor of GIPC differentiation by targeting KLF8. This finding supplied a new target and possible mechanism for the basic research of meat quality improvement.

## Data Availability

The raw data supporting the conclusions of this article will be made available by the authors, without undue reservation.
